# Influence of Steel Fiber Content on the Long-Term Stability of Slag-Containing UHPC Under Different Environments

**DOI:** 10.3390/ma18051068

**Published:** 2025-02-27

**Authors:** Gaoyu Liao, Yikui Xu, Dianchao Wang, Linmei Wu

**Affiliations:** 1 College of Civil Engineering and Architecture, Hunan Institute of Science and Technology, Yueyang 414006, China; gaoyu.liao@hnist.edu.cn (G.L.); chancdp7@gmail.com (Y.X.); 2 Department of Architecture, Graduate School of Engineering, The University of Tokyo, Hongo 7-3-1, Tokyo 113-8654, Japan; wdc@g.ecc.u-tokyo.ac.jp; 3 Department of Mechanical Engineering, University of Western Australia, Perth, WA 6009, Australia

**Keywords:** workability, dimensional stability, fiber-reinforced concrete, compressive strength, underwater engineering

## Abstract

Ultra-high-performance concrete (UHPC) is an advanced material known for its high strength and durability. This study investigates the long-term stability of slag-containing UHPC exposed to outdoor, tap water, and seawater environments over 720 days, focusing on the impact of steel fiber content. The results show that UHPC in tap water exhibits higher compressive strength compared to seawater and outdoor environments. Without fibers, compressive strengths at 1 day, 28 days, and 720 days are 99.7 MPa, 104.7 MPa, and 148.0 MPa, respectively. With 3% steel fiber, these values increase to 132.9 MPa, 143.6 MPa, and 166.6 MPa, representing increases of 33.3%, 37.2%, and 12.6%. Steel fibers also reduce the expansion rate of UHPC, with the expansion decreasing from 525 × 10^−6^ (no fibers) to 345 × 10^−6^ (3% fibers), a reduction of 34.3%. Additionally, differential thermogravimetry (DTG) analysis further confirms the formation of C-S-H, ettringite, calcium hydroxide, and calcite under different environments.

## 1. Introduction

Ultra High-Performance Concrete (UHPC) is an advanced construction material prized for its exceptional strength, durability, and versatility [1]. With compressive strengths exceeding 150 MPa, UHPC offers outstanding mechanical properties, making it ideal for various structural applications. Its dense microstructure provides excellent resistance to wear, abrasion, and corrosion, ensuring long-lasting performance in challenging environments. Moreover, UHPC’s high ductility and flexural strength enable innovative architectural designs and the construction of lightweight structures with reduced material consumption, making it a preferred choice for modern engineering projects worldwide [2,3]. Over the past three decades, UHPC has gained increasing traction in civil engineering circles. However, concerns persist regarding the long-term performance of UHPC under diverse environmental exposures attributable to the abundance of unhydrated cementitious particles within its matrix [2,4]. In marine, outdoor, and water environments, physical alterations and chemical reactions inevitably impact the UHPC structure.

Carbonation is a natural chemical process in which carbon dioxide from the atmosphere reacts with calcium hydroxide and C–S–H in concrete to form calcium carbonate [5]. This reaction gradually penetrates into the concrete’s pore structure, reducing its alkalinity and causing a decrease in pH levels. While carbonation does not typically pose an immediate threat to the structural integrity of concrete, it can lead to corrosion of embedded steel reinforcement over time in the presence of moisture and oxygen [6]. Several studies have addressed carbonated concrete deterioration and proposed formulas to characterize carbonation [7,8,9]. Additionally, corrosion poses a significant concern for UHPC, constituting the primary cause of degradation in reinforced concrete structures. Concrete protects steel bars from corrosion primarily due to its alkaline nature and ability to create a protective environment around the embedded steel. When concrete cures, it forms a highly alkaline pore solution due to the presence of calcium hydroxide. This high pH environment, typically ranging from 12 to 13, creates a passivating layer on the surface of the steel, inhibiting the corrosion process [10,11,12]. However, this passivating state may be compromised by the breakdown of the protective layer resulting from aggressive ion penetration, such as chlorides or environmental acidification near the rebar (carbonation). In marine environments, steel corrosion ensues from chloride ingress into concrete cover. Despite UHPC’s densification compared to conventional concrete, numerous small pores remain, rendering corrosion possible.

In the field of high-strength concrete (HSC), brittleness increases as the concrete strength rises [13]. One approach to mitigate this issue is the incorporation of steel fibers (SFs) into the concrete matrix [14]. Teng et al. [15] observed that using fiber volume fractions of 1.0%, 2.0%, and 3.0%, the 28-day compressive strength of UHPC gradually increased. However, excessive fiber content can negatively affect compressive strength. When the fiber volume fraction exceeds 3.0%, fibers tend to agglomerate, which hinders improvements in compressive strength [16].

Due to its high Portland cement content, the hydration heat of UHPC can be significantly higher compared to conventional concrete. To mitigate this, ground granulated blast furnace slag can be incorporated into the UHPC formulation to reduce Portland cement content, thereby limiting hydration heat while also addressing both ecological and engineering concerns. Yalçınkaya et al. [17] studied the effects of slag replacement at levels of 0%, 30%, and 60% on strength. The results showed that slag replacement resulted in strength levels comparable to those of the reference mixture without slag. Yu et al. [18] investigated the development of eco-friendly UHPCs using efficient mineral admixtures and found that the mechanical properties of UHPC with slag were superior to those of UHPCs containing fly ash or limestone powder. Wu et al. [19] examined the mechanical performance of UHPC mixtures containing slag or fly ash and concluded that the optimal replacement ratios were 40% for slag and 20% for fly ash. Bae and Pyo [20] highlighted that slag can be added to UHPC mixtures to enhance workability and reduce material costs.

Although considerable research has delved into UHPC properties and microstructure, most studies have focused on short-term performance under standard curing conditions [21,22,23,24,25]. Conversely, the literature regarding the long-term behavior of UHPC is limited, particularly in diverse environments encompassing outdoor, tap water, and seawater environments. This study investigates the long-term stability of UHPC in three environments (tap water, seawater, and outdoors) for up to 720 days. This study included compressive strength, mass change rate, dimensional stability, and differential thermogravimetry (DTG). This study is essential to ensure UHPC durability, safety, and economic efficiency in various construction applications.

## 2. Experimental Program

### 2.1. Raw Materials

Portland cement P. I 42.5 (PC) provided by China National Building Materials Co., Ltd. in Beijing, China, adhering to Chinese Standards GB175-2007 [26], was utilized in this study. Table 1 presents a summary of the PC’s physical properties. Blast furnace slag (BFS) obtained from Xiangtan Steelworks in Xiangtan, China, with a specific surface area of 446 m^2^/kg, was employed to replace PC in the UHPC mixture partially. Silica fume (SF) provided by Hunan Ferroalloy Group Co., Ltd. in Xiangxiang, China, characterized by a specific surface area of 18,500 m^2^/kg and a particle size of 0.1~0.5 μm, was also included. Table 2 outlines the chemical composition of the PC, SF, and BFS. River sand, sourced from the Xiang River in Hunan, China, was selected for this study. The sand was sieved to eliminate particles larger than 2.36 mm. The sand has an apparent density of 2550 kg/m^3^, a bulk density of 1570 kg/m^3^, a clay content of 1.0%, and a fineness modulus of 3.0. Steel fiber provided by Hunan Sunshine Steel Fiber Co., Ltd. in Zhuzhou, China, featuring a length of 13 mm, a diameter of 0.2 mm, and a tensile strength of approximately 2800 MPa, was incorporated. A polycarboxylene-based superplasticizer (SP) provided by Sika Building Materials Co., Ltd. in Suzhou, China was utilized, boasting a water-reducing capacity exceeding 30%.

### 2.2. Mix Design

The UHPC mix design was formulated following the methodology established in a prior study [2]. Table 3 details the UHPC mixture proportions. Specifically, a water/binder ratio of 0.18 and a binder/sand ratio of 1:1.1 were chosen and maintained consistently across all experimental batches [1]. The SP was added at a rate of 2% by the binder mass. Steel fiber content was adjusted across different proportions, including 0%, 1%, 2%, and 3% of the concrete volume.

### 2.3. Specimen Preparation

**Figure 1:**
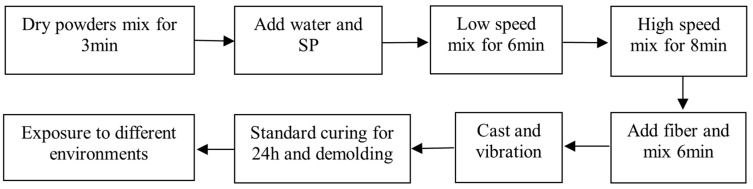
Specimen preparation procedure of UHPC.

The specimen preparation procedure of UHPC is shown in Figure 1. During the mixing process, the dry powder, comprising PC, SF, BFS, and river sand, was initially blended together for 3 min using a mixer. Subsequently, water and SP were introduced and mixed for 6 min at low speed and 8 min at high speed. Following this, steel fibers were added by passing through a sieve with a 5 mm mesh size and mixed for an additional 6 min until uniform distribution was achieved. The fresh UHPC was then poured into molds and subjected to vibration to consolidate the mixture. The specimens were cured in a standard curing room within molds for 24 h, after which they were demolded. Prior to exposure to different environmental conditions, compressive strength, mass, and length of UHPC were measured. Subsequently, the specimens were placed and maintained in various environments until reaching the specified age, following which relevant performance tests were conducted.

### 2.4. Exposure Environments

**Figure 2:**
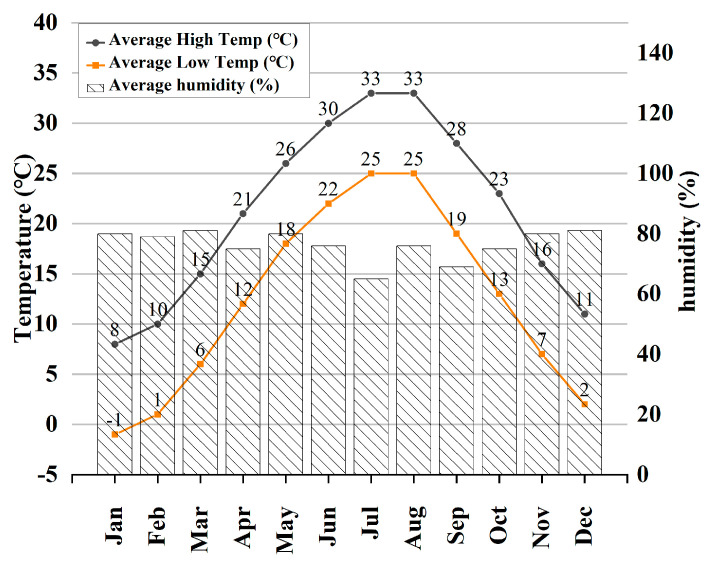
Mean temperature and humidity change in Changsha.

UHPC underwent exposure to three distinct environments over 720 days, which are outlined as follows:

(1) Outdoors: Specimens were placed outdoors in Changsha at a 45° inclination facing south, experiencing natural weather conditions. During this period, the mean temperature and humidity in Changsha, China, are illustrated in Figure 2, fluctuating based on local weather conditions from an average temperature of −1 °C to 33 °C and relative humidity of 76% to 86%.

(2) Seawater: Artificial seawater (AS2) was prepared based on the composition of natural seawater (NS) in the Gulf of China and specifications outlined by ASTM D-14 (AS1) (as presented in Table 4). To expedite the corrosion process over the prolonged exposure period, the concentration of AS2 was increased. Seawater and tap water were refreshed every 3 weeks to ensure a consistent environment.

(3) Tap water: UHPC was submerged in ordinary tap water.

### 2.5. Testing Methods

#### 2.5.1. Workability and Compressive Strength Test

The fluidity test of fresh UHPC was conducted following the procedure outlined in GB/T 2419-2005 [27]. UHPC was exposed in different conditions for 1, 7, 28, 56, 90, 120, 216, 448, 630, and 720 days using the compressive strength test following the guidelines outlined in GB/T 17671-1999 [28]. At each age, a minimum of six specimens underwent testing to establish the average strength.

#### 2.5.2. Dimensional Stability Test

The digital micrometer (BC-300) was used to measure the length change of UHPC in accordance with Chinese standard GB/T50082-2009 [29]. Measurements were conducted at exposure ages of 1, 7, 28, 56, 90, 120, 150, 216, 448, 630, and 720 days.

#### 2.5.3. Mass Change Rate Test

Mass change rate testing methodologies followed the procedures outlined in [30]. An electronic balance was utilized to measure the mass, and the mass change rate was determined using Equation (1):

(1) $$\Delta m=\frac{m_{0}-m_{t}}{m_{0}}$$

where *m*_0_ represents the initial weight and *m_t_* indicates the weight at exposure ages of *t* day.

#### 2.5.4. Differential Thermogravimetry (DTG) Analysis

The sample preparation and DTG testing methodologies followed the procedures outlined in [31,32]. TGA was conducted using the Netzsch STA 409 PC instrument provided by NETZSCH Instruments Company, based in Selb, Bavaria, Germany. The samples were subjected to heating at a rate of 10 °C/min, starting from room temperature to 1000 °C, under a nitrogen atmosphere.

## 3. Results and Discussion

### 3.1. Workability

**Figure 3:**
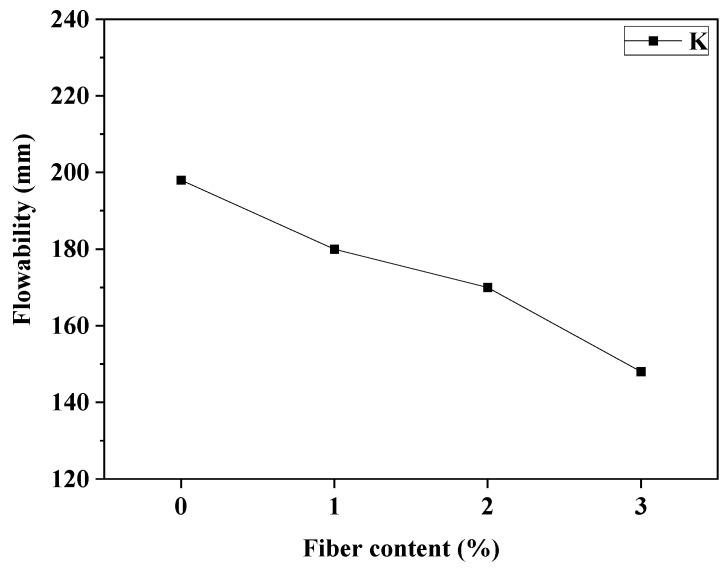
The relationship between fiber contents and flowability of UHPC.

Figure 3 illustrates the UHPC flowability results. Fiber content exerts a pronounced effect on UHPC flowability, with a notable decrease observed as the fiber content increases. This decrease demonstrates a predominantly linear trend. Specifically, as fiber content escalates from 0% to 3%, flowability decreases from 199 mm to 148 mm. The addition of BFS to UHPC further diminishes its flowability. This phenomenon is attributed to the exceedingly high specific surface area of BFS. Notably, BFS particles fill interstices between cement particles, reducing friction among them. Moreover, BFS contributes to a deceleration in the hydration reaction rate of the cementitious system, enhancing porosity and optimizing pore distribution within the matrix.

### 3.2. Compressive Strength

**Figure 4:**
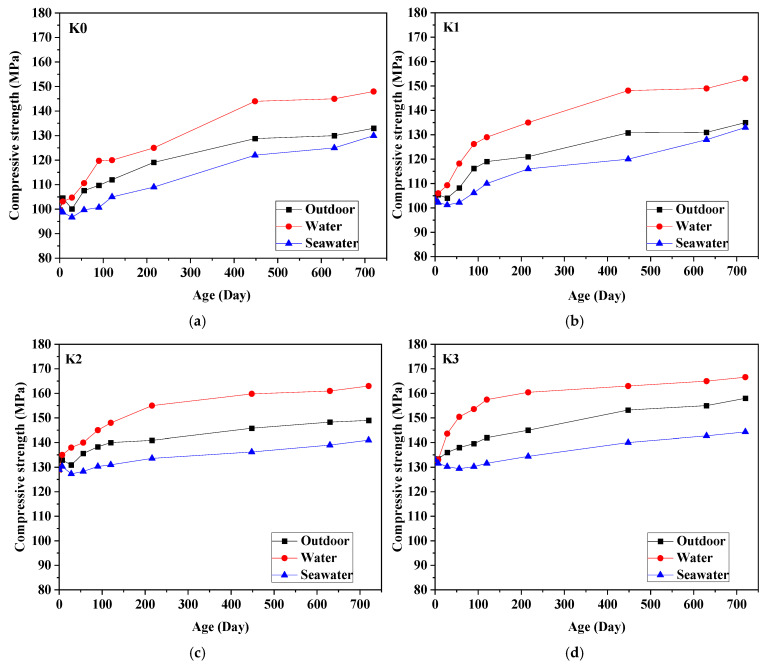
Compressive strength of UHPC exposed to three different conditions. (**a**) K0; (**b**) K1; (**c**) K2; (**d**) K3.

Figure 4 illustrates the variation in the strength of UHPC over time across three distinct curing environments. Generally, early strength development in UHPC is rapid, whereas the late-stage strength increment occurs more gradually. For instance, considering the compressive strength of samples cured in tap water, without the addition of fibers, the compressive strengths at 28 days, 120 days, and 720 days are 104.7 MPa, 120.0 MPa, and 148 MPa, respectively. Compared to the compressive strength at 1 day, which is 99.7 MPa, these values reflect increases of 5.0 MPa (5.0%), 20.3 MPa (20.4%), and 48.3 MPa (48.4%), respectively. With a fiber content of 1%, the compressive strengths of UHPC at 28 days, 120 days, and 720 days are 109.3 MPa, 129.0 MPa, and 153 MPa, respectively. These values represent increases of 5.9 MPa (5.7%), 25.6 MPa (24.8%), and 49.6 MPa (48.0%), respectively, compared to the 1 d compressive strength of 103.4 MPa. For a fiber content of 2%, the compressive strengths at 28 days, 120 days, and 720 days are 138.0 MPa, 148.0 MPa, and 163.0 MPa, respectively. In comparison to the 1 d compressive strength of 129.0 MPa, these values indicate increases of 9.0 MPa (7.0%), 19.0 MPa (14.7%), and 34.0 MPa (26.4%), respectively. With a fiber content of 3%, the compressive strengths at 28 days, 120 days, and 720 days are 143.6 MPa, 157.4 MPa, and 166.6 MPa, respectively. These values show increases of 10.7 MPa (8.1%), 24.5 MPa (18.4%), and 33.7 MPa (25.4%), respectively, compared to the 1-day compressive strength of 132.9 MPa. Across all fiber content levels, the most significant improvements in compressive strength at 28 days, 120 days, and 720 days are observed with a 1% fiber content, while the enhancements are relatively minor with 2% and 3% fiber content.

Furthermore, at the same age, the inclusion of steel fibers from 0% to 3% exhibits an upward trajectory in the compressive strength of UHPC. For instance, considering the strength within tap water curing conditions, in the absence of fibers, the compressive strengths at 1 day, 28 days, and 720 days are 99.7 MPa, 104.7 MPa, and 148 MPa, respectively. However, with a 3% fiber content, the compressive strengths of UHPC are 132.9 MPa, 143.6 MPa, and 166.6 MPa at the same age. These figures represent increases of 33.2 MPa, 38.9 MPa, and 18.6 MPa, respectively, with corresponding percentage increments of 33.3%, 37.2%, and 12.6%. Consequently, the incorporation of steel fibers has significantly enhanced the strength development of UHPC.

Furthermore, upon scrutinizing the evolution of UHPC strength over time across three distinct environments—outdoor, tap water, and seawater—a discernible pattern emerges. Notably, the strength of UHPC within tap water environments demonstrates continuous growth in the later stages. Irrespective of the quantity of steel fiber incorporated, its strength development consistently surpasses that of UHPC in outdoor and seawater environments. This phenomenon primarily stems from the presence of a limited number of interconnected pores within UHPC, enabling water ingress and subsequent hydration of unreacted cement particles to form C-S-H gel, thus enhancing matrix density and strength.

While the strength of UHPC cured outdoors exhibits an incremental trend, it progresses relatively sluggishly compared to specimens cured in water. Conversely, the early strength development of UHPC in seawater environments is gradual, with later-stage strength showing a slow and eventual stabilization at significantly lower levels than those observed in outdoor and tap water environments. This stagnation in strength evolution during later stages can be primarily attributed to two factors. Firstly, the exceedingly low water-to-cement ratio impedes the availability of free water necessary for continued cement hydration and C-S-H gel formation [33]. Secondly, the strength of concrete in later stages is influenced by the reaction between volcanic ash and the hydration product calcium hydroxide (CH). In standard curing conditions, the degree of this reaction between SF and CH is less pronounced than in thermal curing environments.

### 3.3. Dimensional Stability

**Figure 5:**
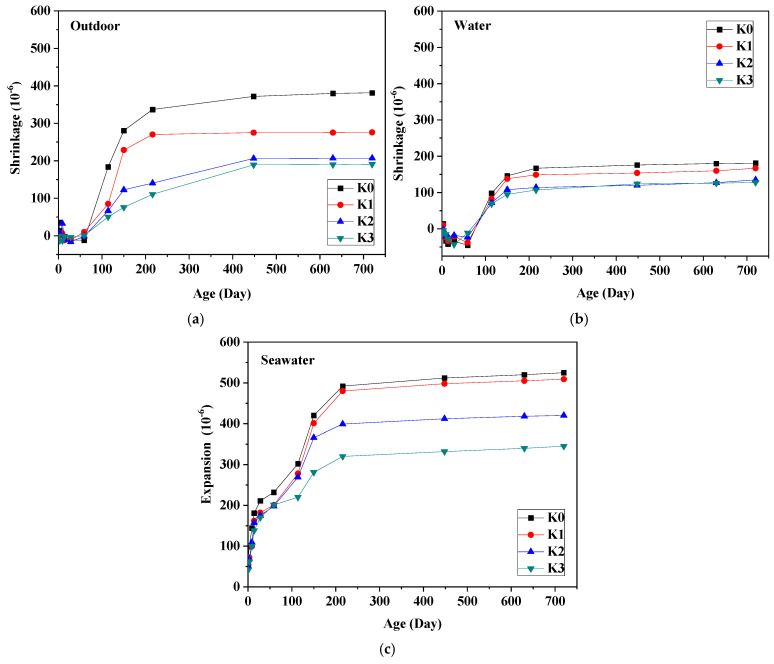
Length change of UHPC. (**a**) Outdoors; (**b**) water; (**c**) seawater.

Figure 5 depicts the length change of UHPC. Notably, UHPC in outdoor and tap water environments exhibit a shrinkage tendency, whereas those exposed to seawater display an expansion trend. Generally, shrinkage stabilizes beyond 150 days of aging. At nearly equivalent ages, the specimens reach their peak strengths, indicating a direct correlation between shrinkage and strength development [34]. Across all conditions, the beneficial effect of steel fiber in stabilizing dimensions is evident [35].

In Figure 5a, the impact of steel fiber content on dimensional changes of UHPC in outdoor conditions is elucidated. The shrinkage rate of each sample group gradually increases with curing age, eventually reaching a stable state. Notably, UHPC without steel fibers exhibits the highest shrinkage rate. Conversely, as steel fiber content rises, the UHPC shrinkage significantly diminishes. For instance, while the shrinkage rate of UHPC without steel fibers after 720 days reaches 381.5 × 10^−6^, the addition of 1%, 2%, and 3% fibers reduces this rate to 276 × 10^−6^, 207.2 × 10^−6^, and 191 × 10^−6^, respectively. Consequently, the reductions are 105.5 × 10^−6^, 174.3 × 10^−6^, and 190.5 × 10^−6^, corresponding to reduction rates of 27.7%, 45.7%, and 49.9%, respectively.

Figure 5b illustrates the impact of steel fiber content on the dimensional variations of UHPC in a tap water environment. The change in shrinkage rate trend closely resembles that observed under outdoor conditions, albeit with markedly lower shrinkage rates than those of specimens exposed to outdoor conditions. For instance, after 720 days, the shrinkage rates for UHPC samples with 0%, 1%, 2%, and 3% fiber content in a tap water environment were 181 × 10^−6^, 167 × 10^−6^, 135 × 10^−6^, and 128 × 10^−6^, respectively. These figures denote reductions of 52.6%, 39.5%, 34.8%, and 33.0% compared to outdoor conditions, respectively. Hence, UHPC specimens in tap water exhibit superior dimensional stability than outdoor specimens. Furthermore, it is evident from the figure that steel fibers mitigate the shrinkage of UHPC. With an increase in steel fiber content, the shrinkage rate of UHPC experiences a significant decrease. For instance, compared to UHPC without steel fibers, the addition of 1%, 2%, and 3% fiber reduces the shrinkage rate by 14 × 10^−6^, 46 × 10^−6^, and 53 × 10^−6^, respectively, after 720 days. This corresponds to reduction rates of 7.7%, 25.4%, and 29.3%, respectively.

The inhibition of shrinkage changes in UHPC samples by steel fibers can be attributed to several factors. Firstly, this phenomenon arises due to the disparity in relative humidity between the external environment and the UHPC system. The incorporation of steel fibers acts akin to providing numerous small supports within the cement paste, preemptively applying to prestress to resist compression. Secondly, steel fibers influence the pore structure, consequently impacting the sample’s length variation. In UHPC specimens lacking steel fibers, more capillary pores form compared to those with steel fibers, resulting in a more refined pore structure. This refinement of pore structure yields both positive and negative effects on length changes [34]. On one hand, it accelerates the rate of reduction of the critical radius, leading to faster shrinkage. On the other hand, it decelerates the migration speed of the water, causing the shrinkage rate controlled by moisture migration to gradually decrease [36]. Consequently, the shrinkage rate is faster at an early age, followed by a gradual slowdown.

Figure 5c illustrates the UHPC dimensional changes in the seawater. Generally, UHPC length in each group exhibits gradual increases with curing age, ultimately stabilizing. Unlike the preceding exposure conditions, UHPC specimens in seawater manifest a tendency to expand. This phenomenon can be attributed to the prolonged soaking period, during which certain substances from seawater gradually permeate into the concrete, generating hydration products such as ettringite and brucite, consequently resulting in lengthening. Santhanam et al. [37] reported a 1.25% expansion in seawater conditions for 32 weeks, associating this expansion with the formation of ettringite. The quantity of brucite fluctuates between 2 and 3%, while the amount of CH decreases steadily. Nagataki et al. [38] observed expansion in concrete attributed to the formation of ettringite and Friedel’s salt resulting from the ingress of chloride and sulfates into the matrix.

Moreover, the inclusion of steel fibers serves to diminish the expansion rate of UHPC. As the quantity of steel fiber increases, the expansion rate of UHPC gradually diminishes. For instance, the expansion rate of specimens without steel fiber after 720 days reached 525 × 10^−6^. Conversely, the expansion rates after adding 1%, 2%, and 3% fiber were 509 × 10^−6^, 420 × 10^−6^, and 345 × 10^−6^, respectively. This corresponds to reductions in expansion rates of 16 × 10^−6^, 105 × 10^−6^, and 180 × 10^−6^, with reduction rates of 3.0%, 20.0%, and 34.3%, respectively.

### 3.4. Mass Change Rate

**Figure 6:**
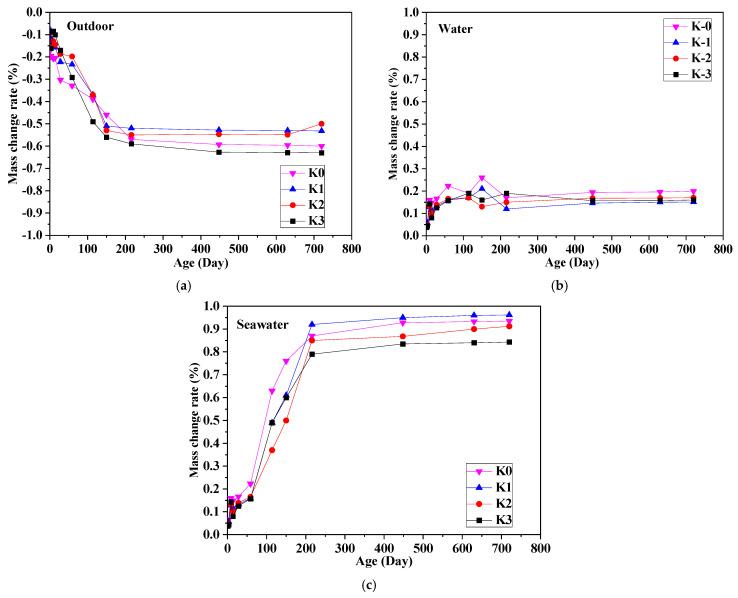
Mass change rate of UHPC. (**a**) Outdoors; (**b**) water; (**c**) seawater.

Figure 6 displays the mass change observed in specimens subjected to three distinct environmental conditions. The findings indicate a correlation between the variations in length and mass of UHPC specimens over exposure time attributable to progressive hydration of the cementitious binder [39]. Notably, a trend of mass reduction is evident in outdoor conditions, while specimens exposed to seawater and tap water environments exhibit a trend of mass increase. Particularly, the mass of specimens immersed in seawater demonstrates a greater increase compared to those in tap water environments.

Figure 6a illustrates the mass change rate of UHPC with varying steel fiber content under outdoor exposure conditions. It was observed that with increasing exposure time, the mass change rate of UHPC gradually escalated, particularly displaying rapid increments within the initial 150 days. Eventually, the final mass change rate stabilized within the range of 0.5% to 0.63%. Notably, the inclusion of steel fibers notably mitigates the early mass change rate of UHPC. However, there is no evident direct correlation between the later mass change rate and the fiber content.

Figure 6b presents the mass change rate of UHPC with varying steel fiber content under tap water immersion conditions. As depicted in the figure, an increasing trend in UHPC mass is evident with prolonged soaking time. After surpassing 28 days, the mass growth rate of UHPC stabilizes within the range of 0.15% to 0.20%. Notably, the addition of steel fiber does not exert a significant effect on the mass growth rate of UHPC under these conditions.

The mass changes of UHPC under various steel fiber content levels under seawater immersion conditions are depicted in Figure 6c. As illustrated, with the duration of immersion increasing, the mass of UHPC exhibits rapid augmentation before 216 days, reaching levels of 0.79% to 0.92%. Eventually, the final mass growth rate stabilizes within the range of 0.843% to 0.962%. This phenomenon primarily arises from the heightened production of ettringite and brucite [37]. Interestingly, as the steel fiber content increases, the mass growth rate during the later period diminishes compared to samples without steel fiber. It is worth noting that the mass change of UHPC in these conditions is significantly smaller than that reported for conventional concrete earlier (ranging from 2% to 12%) [40,41].

### 3.5. TG Analyses

**Figure 7:**
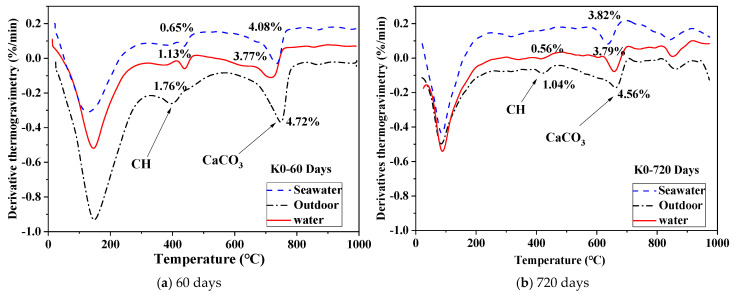
DTG curves of K0 under different environments: (**a**) 60 days; (**b**) 720 days.

Figure 7 displays the DTG curve of UHPC exposure in various conditions for 60 and 720 days. From low to high temperatures, the endothermal peaks were observed within three distinct temperature ranges, corresponding to the dehydration of C-S-H, ettringite, and evaporable water, dehydroxylation of calcium hydroxide, and decomposition of calcium carbonate, respectively [42,43].

At 60 days, notable differences are observed in the first endotherm among samples subjected to outdoor conditions, indicating a greater mass loss with significantly stronger intensity compared to the other two conditions. This suggests that the UHPC in outdoor conditions contained a higher proportion of hydration product phase, including C-S-H and ettringite. In contrast, it decreased in UHPC immersed in water and seawater. The slightly elevated temperature associated with the water evaporation in samples exposed to outdoor conditions indicates a tighter bond with the phases. Moreover, the prominent peak attributed to the dehydroxylation of calcium hydroxide indicates that UHPC exposed to outdoor conditions experienced fewer phase changes compared to those in the other two conditions, despite some conversion of calcium hydroxide to calcium carbonate due to carbonation effects.

The UHPC immersed in seawater exhibits the smallest peak, indicating a significant loss of C-S-H gel due to exposure to seawater. This loss is likely due to the reaction between C-S-H and various ions in seawater, particularly sulfates. At 720 days, the content of CH in UHPC cured in seawater diminishes more rapidly compared to UHPC cured in other environments. This reduction in CH content over time may be associated with the consumption of volcanic ash reaction products from BFS. The leached calcium hydroxide reacts with Mg^2+^ and SO_4_^2−^ in seawater, forming brucite and ettringite.

The concentration gradient induced by the reaction further facilitates leaching, perpetuating the formation of brucite over time. Additionally, soluble carbonate ions present in seawater can trigger a reaction with calcium hydroxide to precipitate calcite on the UHPC surface. Consequently, the leaching rate of calcium hydroxide is highest in UHPC exposed to seawater.

The calcium hydroxide content of UHPC exposed to tap water exceeds that of seawater-exposed specimens but falls below that of specimens in outdoor conditions. Additionally, its CaCO_3_ content is lower than that of specimens in seawater and outdoor conditions. Furthermore, the calcium hydroxide content decreases from 60 to 720 days. This phenomenon arises from the reaction of calcium hydroxide formed during cement hydration with BFS to produce C-S-H and its reaction with soluble carbonate ions in water to form CaCO_3_.

UHPC in outdoor exhibits the highest levels of calcium hydroxide (CH) and CaCO_3_. This is attributed to moisture loss during outdoor exposure, preventing the CH formed during cement hydration from reacting with BFS. The elevated CaCO_3_ content results from the reaction of atmospheric carbon dioxide with CH. Notably, the carbonization rate of UHPC is minimal [44], primarily occurring on the surface.

Given the negligible difference in UHPC composition, it is evident that leaching and carbonization of CH are the primary factors contributing to its consumption within the matrix. According to Carde et al. [45], when the binder encounters water, ion migration occurs between the soluble matrix and the external water, disrupting the chemical equilibrium of the hydrates and medium. However, once hydrates dissolve or precipitate, equilibrium is restored [46,47]. CH is the initial mineral that dissolves, followed by the gradual decalcification of C-S-H. Furthermore, CH formed by cement hydration in UHPC specimens cured in seawater and tap water can react with BFS to produce C-S-H. This reaction improves the pore structure of the specimen, thereby enhancing its mechanical properties and impermeability.

## 4. Conclusions

This study comprehensively analyzed the influence of steel fiber content and various environmental exposures on the long-term stability of UHPC, encompassing changes in strength, length, and mass, while also examining their impact on the microstructure of UHPC. The key conclusions drawn are as follows:

(1) An increase in steel fiber content led to a reduction in the flowability of fresh UHPC.

(2) The strength of UHPC in tap water environments exhibited continuous growth in later stages, consistently surpassing the strength of UHPC in seawater and tap water environments. Specimens cured in seawater exhibited lower strength compared to those cured outdoors. Furthermore, higher steel fiber content is generally correlated with increased UHPC strength.

(3) Regarding length change, UHPC exposed to outdoor and tap water experienced shrinkage, with outdoor specimens exhibiting greater shrinkage compared to those cured in water. Conversely, specimens exposed to seawater displayed expansion behavior. Notably, the inclusion of steel fibers, particularly at 2% and 3%, mitigated length changes.

(4) Mass changes varied across exposure conditions: outdoor-exposed specimens exhibited mass loss due to moisture evaporation, tap water-exposed specimens showed a slight mass increase, and seawater-exposed specimens demonstrated mass gain. The utilization of BFS and the addition of fibers did not significantly influence mass changes.

(5) DTG analysis revealed that UHPC immersed in seawater effectively consumed CH over 720 days. Reduction in CH was accompanied by the formation of calcium carbonate under outdoor conditions due to carbonization. In seawater environments, CH transformed into brucite and ettringite. Additionally, early loss of C-S-H gel was observed in seawater and tap water environments, particularly after 60 days of immersion.

While this study provides valuable insights into the long-term stability of slag-containing UHPC under various environmental conditions, there are several limitations. The study primarily focused on the effects of steel fiber content and environmental exposure without exploring other potential additives or mix designs that may further enhance UHPC’s performance. Additionally, the experimental duration of 720 days may not fully capture the long-term behavior of UHPC in extreme environments. Future research could explore the influence of other fibers or supplementary cementitious materials, such as metakaolin, on the performance of UHPC. Moreover, extending the duration of exposure and including additional environmental conditions, such as freeze–thaw cycles, could provide a more comprehensive understanding of UHPC’s long-term durability.

## Figures and Tables

**Table 1:** Physical properties of PC.

Density (kg/m^3^)	Specific Surface Area (m^2^/kg)	80 μm Residue on Sieve (%)	Setting Time (Hour)		Flexural Strength (MPa)		Compressive Strength (MPa)	
			Initial	Final	3 Days	28 Days	3 Days	28 Days
3.15	380	0.3	2.5	3.4	6.4	9.0	33.0	60.0

**Table 2:** Chemical composition of PC, SF, and BFS (%).

Oxide (wt. %)	SiO_2_	CaO	Al_2_O_3_	MgO	Fe_2_O_3_	Na_2_O	K_2_O	SO_3_	LOI
PC	25.26	64.67	6.38	2.68	4.05	-	-	0.94	0.9
SF	90.82	0.45	1.03	0.83	1.50	0.17	0.86	-	4.34
BFS	33.00	39.11	13.91	10.04	0.82	0.26	1.61	0.92	0.33

**Table 3:** UHPC mixture proportions.

No.	w/b	Mass (kg/m^3^)						
		Water	PC	SF	BFS	SP *	Fiber	Sand
K0	0.18	164	550	200	250	20	0.0	1100
K1	0.18	164	550	200	250	20	78.5	1100
K2	0.18	164	550	200	250	20	157.0	1100
K3	0.18	164	550	200	250	20	235.5	1100

* SP means total mass of liquid liquid-based SP.

**Table 4:** The compositions of NS, AS1, and AS2 (g/L).

Chemical	NaCl	MgCl_2_	Na_2_SO_4_	MgSO_4_·7H_2_O	MgCl_2_·6H_2_O	CaSO_4_·2H_2_O	CaCO_3_	CaC1_2_	KCl
NS	21.0	2.54	-	1.54	-	2.43	0.1	-	-
AS1	24.5	-	4.1	-	2.54	-	-	1.2	0.1
AS2	25.0	12.7	10.0	-	7	-	-	-	0.1

## Data Availability

The original contributions presented in this study are included in the article. Further inquiries can be directed to the corresponding author.
